# Effect of weight on depression using multiple genetic instruments

**DOI:** 10.1371/journal.pone.0297594

**Published:** 2024-02-23

**Authors:** Jutta Viinikainen, Petri Böckerman, Barton Willage, Marko Elovainio, Jaana T. Kari, Terho Lehtimäki, Jaakko Pehkonen, Niina Pitkänen, Olli Raitakari

**Affiliations:** 1 Jyväskylä University School of Business and Economics, University of Jyväskylä, Jyväskylä, Finland; 2 Labour Institute for Economic Research LABORE, Helsinki, Finland; 3 IZA Institute of Labor Economics, Bonn, Germany; 4 Department of Economics, University of Colorado—Denver, Denver, Colorado, United States of America; 5 Department of Psychology and Logopedics, University of Helsinki, Helsinki, Finland; 6 Finnish Institute for Health and Welfare, Helsinki, Finland; 7 Department of Clinical Chemistry, Fimlab Laboratories, Tampere, Finland; 8 Faculty of Medicine and Health Technology, Tampere University, Tampere, Finland; 9 Finnish Cardiovascular Research Center Tampere, Tampere University, Tampere, Finland; 10 Research Centre of Applied and Preventive Cardiovascular Medicine, University of Turku, Turku, Finland; 11 Centre for Population Health Research, University of Turku and Turku University Hospital, Turku, Finland; 12 Department of Clinical Physiology and Nuclear Medicine, Turku University Hospital, Turku, Finland; University of Botswana School of Medicine, BOTSWANA

## Abstract

A striking global health development over the past few decades has been the increasing prevalence of overweight and obesity. At the same time, depression has become increasingly common in almost all high-income countries. We investigated whether body weight, measured by body mass index (BMI), has a causal effect on depression symptoms in Finland. Using data drawn from the Cardiovascular Risk in Young Finns Study (N = 1,523, mean age 41.9, SD 5), we used linear regression to establish the relationship between BMI and depression symptoms measured by 21-item Beck’s Depression Inventory. To identify causal relationships, we used the Mendelian randomization (MR) method with weighted sums of genetic markers (single nucleotide polymorphisms, SNPs) as instruments for BMI. We employ instruments (polygenic risk scores, PGSs) with varying number of SNPs that are associated with BMI to evaluate the sensitivity of our results to instrument strength. Based on linear regressions, higher BMI was associated with a higher prevalence of depression symptoms among females (b = 0.238, p = 0.000) and males (b = 0.117, p = 0.019). However, the MR results imply that the positive link applies only to females (b = 0.302, p = 0.007) but not to males (b = -0.070, p = 0.520). Poor instrument strength may explain why many previous studies that have utilized genetic instruments have been unable to identify a statistically significant link between BMI and depression-related traits. Although the number of genetic markers in the instrument had only a minor effect on the point estimates, the standard errors were much smaller when more powerful instruments were employed.

## Introduction

In recent decades, there has been a notable increase in the prevalence of overweight and obesity, while at the same time, depressive disorders have emerged as a leading cause of disease burden worldwide [1–3]. The question of whether increased body weight and increased depression are causally linked has become increasingly pressing. Previous research, primarily from the United States (US), has established a correlation between measures of body weight, such as body mass index (BMI), and depression [4,5], but identifying the direction of causality remains challenging. To draw policy-relevant conclusions, prior studies examining the impact of weight on mental health have used the BMI of a biological relative as an instrument for one’s own BMI [6,7]. These studies found evidence that weight causally affects mental health. Recently, researchers have begun to investigate the causal effects of weight on depression symptoms using genetic information as the instrument. Most of these studies are based on US data and have not found a significant effect of BMI on mental health [8–10]. However, a high BMI has been linked to weaker mental health among the US elderly [11], and evidence from Finland and the United Kingdom suggests that excessive body weight may increase the risk of depression [12,13].

In the European Union (EU), 1 in 6 people experiences a mental health problem. Among these countries, Finland has the highest estimated incidence of mental disorders, with nearly 1 in 5 people affected [14]. Additionally, the proportion of the population classified as overweight (BMI ≥ 25) is higher in Finland (59%) compared to the EU average (53%) [15], and the prevalence of obesity has been increasing [16]. Using data from Finland, this study examines how BMI is linked to depression symptoms among prime working-age individuals. To investigate this research question, we use instruments based on genetic markers, single nucleotide polymorphisms (SNPs), to identify causal effects. Recently, genome-wide association studies (GWASs) have identified an increasing number of SNPs related to BMI. Aggregating multiple weight-predictive SNPs into a single number yields a polygenic risk score, (PGS) which indicates an individual’s genetic susceptibility to high BMI. A higher number of SNPs in a PGS enhances the strength of the instrument, which mitigates the potential weak instrument problem associated with the statistical approach we employ. However, it also amplifies the risk that the SNPs affect the outcome through pathways other than BMI, thereby violating the key identifying assumptions. Consequently, the secondary aim of this study is to compare estimates using different instruments to shed light on the sensitivity of the results to which instruments are used.

## Materials and methods

### Study sample

The Cardiovascular Risk in Young Finns Study (YFS) is an ongoing study that began in 1980 with a total of 3,596 participants between 3 and 18 years of age. The participants were randomly chosen from five Finnish university regions [17]. The main goal of the study was to determine the contribution of childhood lifestyle, biological, and psychological measures to the risk of cardiovascular diseases in adulthood. Risk factors associated with cardiovascular disease, such as information on BMI, have also been repeatedly collected from the survey participants. Moreover, information on psychosocial traits has been collected over the study.

**Fig 1:**
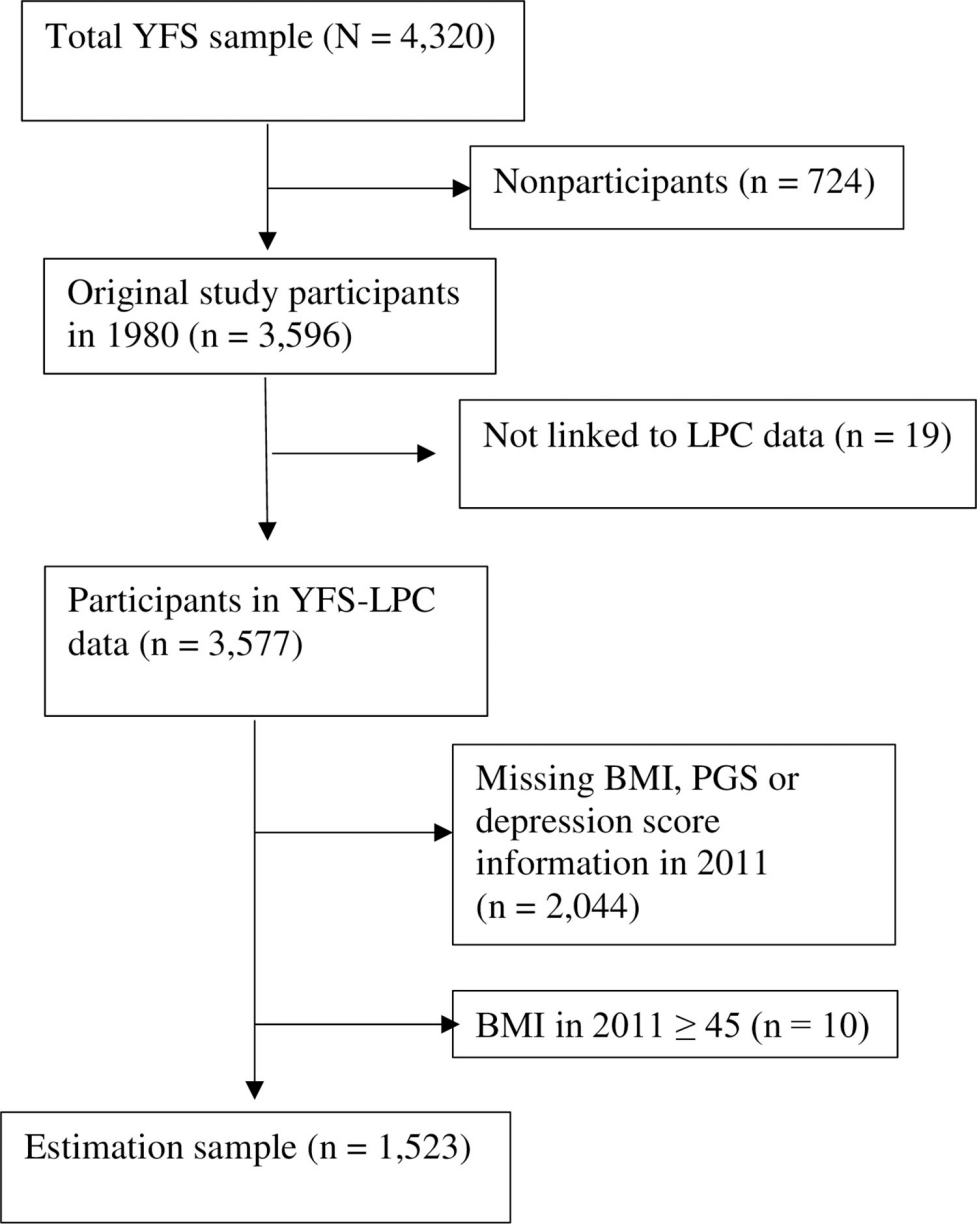
Flow chart of the YFS-LPC data. Participants in the YFS who were not linked to the LPC data either did not consent to the linking or were not found in the registers, due to having permanently moved abroad or having passed away before 1987.

We perform secondary analysis using YFS data from 2011, i.e., 31 years after the study was launched when the participants were, on average, 41.9 years of age (SD = 5 years). To obtain information on participants’ parental background, the YFS was linked to the Longitudinal Population Census (LPC) of Statistics Finland from the year 1980 using unique personal identifiers. The current analysis is based on a sample size of 1,523. The reduced size compared to the original sample is mainly due to missing information for certain variables. Fig 1 depicts a flowchart of the study sample showing the data sources and the exclusion criteria of individuals at each stage.

The YFS was approved initially by the ethical committee of the University of Turku (3/1978) and subsequently by the ethical committee of the Hospital District of Southwest Finland whenever a new field study was conducted, most recently in 2017 (ETMK 68/2017). The use of the YFS-LPC data was approved by Statistics Finland (TK-53-673-13). Written informed consent for underage participants was provided by their parents or guardians. Once participants reached the age of 18, they themselves gave written informed consent after a full explanation of the study procedures.

### Measures

As the dependent variable, we used the 21-item Beck’s Depression Inventory (BDI-II), which was administered in 2011 [18,19]. Items include feeling sad, feeling discouraged about the future, and feelings of failure. S1 Table documents the full inventory. Depression symptoms were self-assessed on a 4-point scale, and the total score ranges from 0 to 63. A higher value of the score indicates more severe depression symptoms.

As the treatment variable, we used BMI, which is defined as weight in kilograms over height in meters squared (kg/m^2^). BMI originated from the YFS data and was based on professional health examinations conducted in 2011. Since body measures were collected by health professionals, we avoid measurement errors and other sources of bias related to self-reported BMI [20].

Information on family background, measured by parental education, was obtained in 1980 (from LPC). The parental education indicator equaled one if the parent had completed university level education by 1980 and zero otherwise. Information on region of residence were measured in 1980, and the variable takes one of four categorical values (south, west, east, north, from YFS).

Blood samples from the YFS participants, collected in 2001 or in 2007, were used for genotyping. Based on the genotyping results, SNPs associated with BMI in two GWASs were identified [21,22]. We use weighted PGSs as instruments, where the weights are determined by the strength of the SNP’s association with BMI. The sum of these weighted contributions yields the PGS. The first PGS, which has also been used in previous studies [8–10], includes 32 SNPs that are associated with BMI at p < 5×10^−8^ [21]. Moreover, we use five PGSs based on a more recent GWAS [22]. These PGSs include SNPs that are associated with BMI at the following significance levels: p < 5×10^−8^, p < 10^−5^, p < 10^−4^, p < 0.001, and p < 0.01. The PGS based on significance level p < 5×10^−8^ includes 97 SNPs. In other PGSs, the number of SNPs is higher, but the exact number is unknown.

### Statistical analysis

We first ran an OLS model that shows an association between BMI and depression symptoms. To identify a causal effect of BMI on depression symptoms, we then estimated two-stage least squares (2SLS), Mendelian randomization (MR) models, using PGSs as instruments for BMI. The motivation for the use of MR is based on the likely influence of confounding factors [5,10], and the possibility of a two-way causal relationship between body weight and depression [23,24]. The analyses were conducted using Stata, version 18.0.

In all models, we controlled for age, sex, region of residence in 1980, and parental education. To account for population stratification, the MR models also include the first ten genetic principal components.

To obtain unbiased MR estimates, four conditions must be met: (1) the genetic instrument must be as-if randomly assigned (*independence*); (2) the instrument must have a monotonic effect on BMI (*monotonicity*); (3) the genetic instrument must affect BMI (*relevance*); and (4) the genetic instrument does not affect depression except through BMI (*exclusion restriction*) [11].

The independence assumption is supported by Mendel’s law of segregation i.e., alleles segregate randomly in conception. To control for the possibility that the independence assumption would be violated because of population stratification, which is the case if SNP frequencies differ between population subgroups, we control for the first ten genetic principal components. Supporting the monotonicity assumption, the SNPs in PGSs are associated with higher (not lower) BMI. To evaluate the relevance assumption, it has become standard to use the first-stage statistics F-value of 10 as a cut-off value for a sufficiently strong instrument [25]. However, a recent study suggests that this cut-off value may be too low and proposes that the first-stage F-statistics should exceed the value of 104.7 [26]. In the studies that use the 32 SNP genetic instrument, the first-stage F-statistics have been around 35 [8–10], which clearly falls below the more conservative cut-off value. In this study, we also use an instrument that exceeds the cut-off value of 104.7. The exclusion restriction assumption is discussed in the results section.

## Results

**Fig 2:**
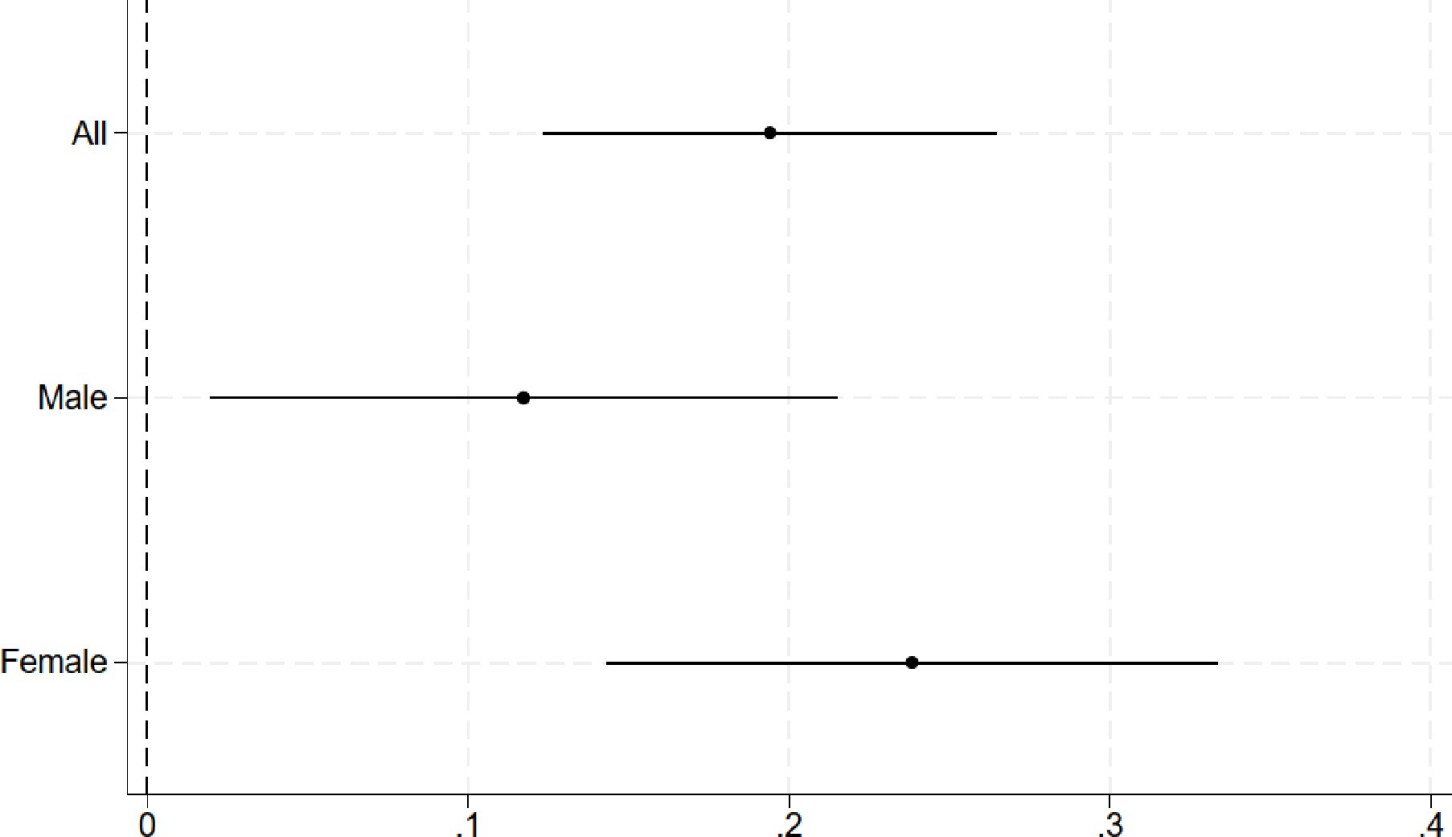
The association between BMI and depression symptoms based on OLS estimates. OLS point estimates (points) and the corresponding 95% confidence intervals (solid lines) based on heteroscedasticity-robust standard errors overall and stratified by sex. The models adjust for (sex), age, region of residence in 1980, and parental education. N = 1,523.

Descriptive statistics of the variables are presented in Table 1. The OLS estimates in Fig 2 and Table 2 (Panel A) show that a one-unit increase in BMI is associated with a 0.117 [95% CI: 0.020, 0.215] and 0.238 [95% CI: 0.143, 0.333] unit increase in the depression index among males and females, respectively.

**Table 1:** Descriptive statistics.

	Data source	Year of measurement	Mean	SD
Beck’s Depression Inventory score	YFS	2011	4.991	5.784
BMI	YFS	2011	26.275	4.677
BMI PGS, Speliotes *p*<5×10^−8^	YFS	2001/2007	4.030	0.527
BMI PGS, Locke *p*<5×10^−8^	YFS	2001/2007	-0.301	0.191
BMI PGS, Locke *p*<10^−5^	YFS	2001/2007	-0.409	0.266
BMI BGS, Locke *p*<10^−4^	YFS	2001/2007	-0.467	0.333
BMI PGS, Locke *p*<0.001	YFS	2001/2007	0.595	0.452
BMI PGS, Locke *p*<0.01	YFS	2001/2007	0.964	0.737
Female, share	YFS	1980	0.550	0.498
Average age, years	YFS	2011	41.861	5.039
Mother education high, share	LPC	1980	0.077	0.267
Father education high, share	LPC	1980	0.106	0.308
Region of residence, share				
Southern Finland	YFS	1980	0.169	0.375
Western Finland	YFS	1980	0.357	0.479
Eastern Finland	YFS	1980	0.309	0.462
Northern Finland	YFS	1980	0.165	0.372

YFS refers to the Cardiovascular Risk in Young Finns Study. LPC refers to the Longitudinal Population Census. SD refers to standard deviation and PGS refers to polygenic risk score. The PGSs are based on studies by Speliotes et al. (2010) and Locke et al. (2015). The indicator for high parental education equals one if the parent has obtained some university education based on the LPC data from 1980. N = 1,523.

**Table 2:** Estimation results: BMI and depression symptoms.

	All	Male	Female
Panel A: OLS results			
Per additional unit of BMI	0.194*** (p = 0.000) [0.123, 0.265]	0.117** (p = 0.019) [0.020, 0.215]	0.238*** (p = 0.000) [0.143, 0.333]
Panel B: MR results (PGS by Speliotes, p < 5×10^−8^ (32 SNPs)			
Per additional unit of BMI	0.235 (p = 0.238) [-0.155, 0.625]	0.228 (p = 0.387) [-0.288, 0.743]	0.276 (p = 0.355) [-0.309, 0.862]
First stage F-statistics	36.45	19.42	16.49
Panel C: MR results, (PGS by Locke, p < 5×10^−8^ (97 SNPs)			
Per additional unit of BMI	0.190 (p = 0.277) [-0.152, 0.531]	0.096 (p = 0.704) [-0.400, 0.592]	0.261 (p = 0.259) [-0.193, 0.714]
First stage F-statistics	48.04	21.93	26.65
Panel D: MR results (PGS by Locke, p < 10^−5^)			
Per additional unit of BMI	0.136 (p = 0.354) [-0.151, 0.423]	-0.052 (p = 0.814) [-0.484, 0.380]	0.256 (p = 0.172) [-0.112, 0.624]
First stage F-statistics	71.27	35.09	37.54
Panel E: MR results (PGS by Locke, p < 10^−4^)			
Per additional unit of BMI	0.182 (p = 0.141) [-0.060, 0.425]	-0.089 (p = 0.659) [-0.485, 0.307]	0.360** (p = 0.018) [0.063, 0.656]
First stage F-statistics	101.07	39.59	60.80
Panel F: MR results (PGS by Locke, p < 0.001)			
Per additional unit of BMI	0.127 (p = 0.242) [-0.086, 0.339]	-0.116 (p = 0.450) [-0.416, 0.185]	0.289* (p = 0.050) [0.000, 0.577]
First stage F-statistics	162.50	73.92	88.44
Panel G: MR results (PGS by Locke, p < 0.01)			
Per additional unit of BMI	0.163 (p = 0.041) [0.007, 0.319]	-0.070 (p = 0.520) [-0.282, 0.143]	0.302*** (p = 0.007) [0.083, 0.521]
First stage F-statistics	285.84	136.42	156.62
Mean outcome	4.991	4.095	5.726
N	1,523	686	837

The table reports parameter estimates, p-values in parentheses and 95% confidence intervals based on heteroscedasticity-robust standard errors in square brackets. The dependent variable is Beck’s Depression Inventory score measured in 2011. BMI was measured in 2011. The models include unreported controls for (sex), age, the first ten principal components (Panels B-G), region of residence in 1980, and parental education. The instruments used in the MR models are the PGSs for BMI based on studies by Speliotes et al. (2010) and Locke et al. (2015) [21,22]. Boldface indicates statistical significance (*p < 0.10

**p < 0.05

***p < 0.001). N = 1,523.

**Fig 3:**
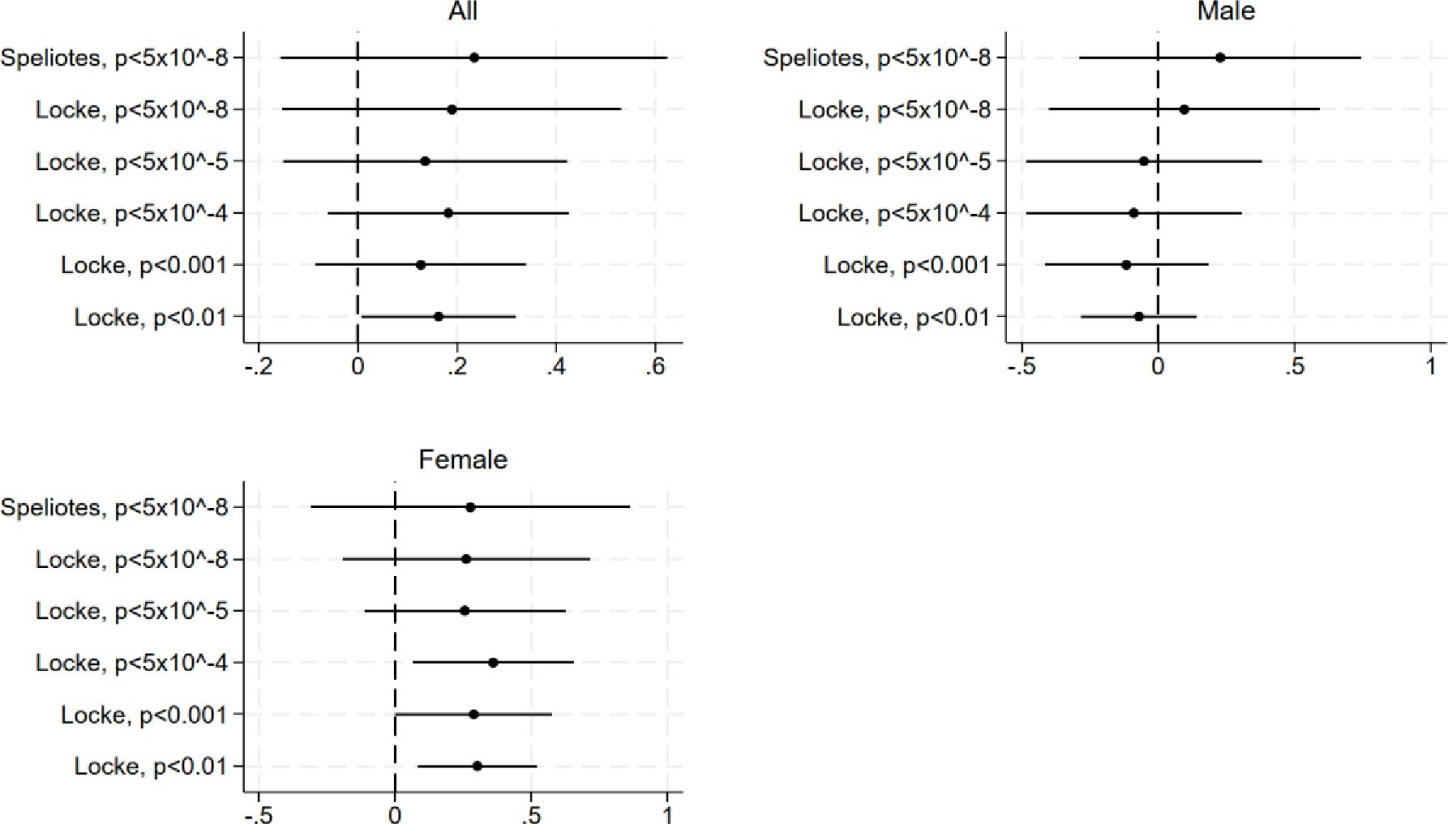
The effect of BMI on depression symptoms, MR estimates. MR point estimates (points) and the corresponding 95% confidence intervals (solid lines) based on heteroscedasticity-robust standard errors overall and stratified by sex. The instruments used in the models are the PGSs for BMI based on studies by Speliotes et al. (2010) and Locke et al. (2015) [21,22]. The PGSs include SNPs that associated with BMI at the following significance levels: p < 5×10^−8^, p < 10^−5^, p < 10^−4^, p < 0.001, and p < 0.01. The models adjust for (sex), age, the first ten principal components, region of residence in 1980, and parental education. N = 1,523.

Fig 3 and Table 2 (Panels B-G) summarize the MR estimates for the effect of BMI on mental health. We obtained three important results. First, the point estimates were similar regardless of the instrument used. Second, the positive link between BMI and depression symptoms applies only to females. The results using the instrument based on p < 0.01 imply that among females a one-point increase in BMI is linked to a 0.302 [95% CI: 0.083, 0.521] unit increase in depression symptoms. Among males, the point estimate is negative, close to zero, and statistically insignificant (b = -0.070, 95% CI: -0.282, 0.143). Results from reduced-form models are in accordance with these results (S1 Fig). Third, the more SNPs the instrument contains, the smaller the confidence intervals. This is important pattern affects the interpretation of the results, particularly for females. Using an instrument that is based on a significance level of p < 10^−5^ or lower, the results suggest a statistically insignificant link between BMI and depression symptoms for both females and males. Using other instruments, the link is positive and significant (p < 0.10) for females. The first-stage F-statistics in the models using instruments based on significance level p < 10^−5^ or lower vary between 16.49 and 71.27. In the models using an instrument based on more lenient significance levels, the F-statistics vary between 39.59 and 285.84.

A potential threat to the validity of the results is the violation of the exclusion restriction assumption. This assumption could be violated if SNPs, which are related to BMI, also affect depression symptoms via other pathways. To assess the validity of our instrument, we conducted a balance test comparing observable characteristics of individuals above- and below- median PGS scores (Table 3). The 32 SNP PGS is not associated with covariates, but the p < 0.01 PGS is associated with residing in eastern or western Finland and fathers’ education, which are controlled in all models. We also performed Sargan’s test of overidentifying restrictions using the 32 SNP PGS. The test lends support to the instrument’s validity as the test supported the null hypothesis that all 32 SNPs yielded the same MR estimate (p > 0.520). As we lack information on individual SNPs for other instruments, Sargan’s test cannot be used to test the validity of those instruments. However, as the point estimates were similar across models, Sargan’s test based on 32 SNP PGS indirectly supports the conclusion that violation of the exclusion restriction does not substantially bias the results.

**Table 3:** Comparison of observable characteristics between individuals above and below the median values of PGS.

	Above median PGS	Below median PGS	Difference^a^ (t-statistics)
Female: Speliotes^c^, p < 5×10^−8^	0.548 (0.498)	0.551 (0.498)	-0.003 (-0.100)
Female: Locke^d^, p < 0.01	0.550 (0.497)	0.549 (0.498)	0.001 (0.023)
Age in 2011: Speliotes, p < 5×10^−8^	41.692 (5.013)	42.033 (5.063)	-0.341 (-1.321)
Age in 2011: Locke, p < 0.01	41.701 (5.088)	42.022 (4.988)	-0.322 (-1.245)
Southern Finland^b^: Speliotes, p < 5×10^−8^	0.163 (0.370)	0.176 (0.381)	-0.013 (-0.650)
Southern Finland^b^: Locke, p < 0.01	0.154 (0.361)	0.185 (0.389)	-0.032 (-1.651)
Western Finland^b^: Speliotes, p < 5×10^−8^	0.349 (0.477)	0.365 (0.482)	-0.016 (-0.653)
Western Finland^b^: Locke, p < 0.01	0.336 (0.473)	0.377 (0.485)	**-0.041 (-1.678)** *
Eastern Finland^b^: Speliotes, p < 5×10^−8^	0.322 (0.468)	0.295 (0.456)	0.028 (1.177)
Eastern Finland^b^: Locke, p < 0.01	0.346 (0.476)	0.271 (0.445)	**0.076 (3.209)** ***
Northern Finland: Speliotes, p < 5×10^−8^	0.166 (0.372)	0.165 (0.372)	0.001 (0.035)
Northern Finland: Locke, p < 0.01	0.164 (0.371)	0.167 (0.373)	-0.003 (-0.149)
High education, mother: Speliotes, p < 5×10^−8^	0.074 (0.263)	0.081 (0.272)	-0.006 (-0.450)
High education, mother: Locke, p < 0.01	0.071 (0.257)	0.084 (0.278)	-0.013 (-0.966)
High education, father: Speliotes, p < 5×10^−8^	0.107 (0.309)	0.106 (0.308)	0.001 (0.087)
High education, father: Locke, p < 0.01	0.089 (0.285)	0.124 (0.329)	**-0.034 (-2.172)** **

The table reports means and standard deviations in parentheses. Boldface indicates statistical significance (*p < 0.10

**p < 0.05

***p < 0.001). N = 1,523.

^a^ A two-sample t-test was used to test the differences in means.

^b^ Regional indicators refer to the region of residence in 1980.

## Discussion

Using genetic instruments for BMI, we found that BMI was linked to depressive symptoms among females but not among males in Finland. Although the point estimates were similar regardless of the PGS used, the confidence intervals were substantially reduced as the instrument strength increased. The first result is consistent with previous findings that females are particularly punished for being overweight, e.g., in the labor market [27,28]. The second finding may explain why previous studies that used a genetic instrument based on relatively fewer SNPs have not found a significant relationship between BMI and depression-related traits. Consistent with prior studies [8–10], our results based on the 32 SNP PGS, did not indicate a significant link between BMI and depressive symptoms. Using a more powerful instrument, the standard errors were reduced, and the link was statistically significant among females. Notably, the earlier studies that have used more powerful PGSs have supported the causal link between BMI and depressive symptoms [11,13]. Our results based on the 32 SNPs PGS contradict prior findings based on YFS data that used a modified version of Beck’s Depression Inventory with a 31 SNP PGS [12]. In this study, we used the original (i.e., unmodified) version of Beck’s inventory.

Potential data limitations include the data collection occurring in five university regions in Finland, therefore, the data may not be fully nationally representative of the total population of Finland. Moreover, attrition occurred over the course of the study. However, importantly, based on earlier results, there has not been attrition with respect to BMI [17]. Our analysis used BMI measures and depression scores from the follow-up that was conducted in 2011. While these measures are a decade old, we believe our analysis is still highly relevant to the current situation. For comparison, using National Health and Nutrition Examination Survey (NHANES) data from the United States, we found that there is very little difference in the raw correlation between BMI and depression between 2005 and 2017 (S2 Table). The most important limitations of this study are related to the validity of the MR assumptions. The use of MR requires that the exclusion restriction is satisfied, and the threat that this assumption is violated increases with the number of SNPs in the PGS. The exclusion assumption is strictly untestable. However, Sargan’s test results were consistent with instrument validity, and the similarity of point estimates regardless of instrument is encouraging. This suggests that the potential violation of the exclusion restriction does not cause meaningful bias in the results.

The main strength of this study is that the BMI information is based on objective measures and our measure of depressive symptoms was based on one of the most widely used measures (BDI-II), which has strong internal consistency and test-retest reliability [29]. Crucially, the availability of PGSs with varying amounts of SNPs enabled us to examine how the strength of the instrument affects the conclusions we draw from the MR analysis.

The World Health Organization (WHO) has stated that the global burden of obesity is a major public health challenge [30]. Obesity is an important risk factor of many noncommunicable diseases, including diabetes and cardiovascular diseases, and the estimated costs associated with increased mortality and morbidity due to obesity are already substantial [31,32]. WHO has set a target of zero growth in the prevalence of obesity between 2010 and 2030, aligning with United Nations’ Sustainable Development Goal 3 “Good Health and Well-being” [30]. Based on our results, achieving this goal may also help prevent depressive disorders, which have significantly increased worldwide [33]. Prior research has shown that social stigma is a likely mechanism between weight and mental health. Therefore, policy interventions that educate the public and reduce stigma toward individuals with higher BMI could help to reduce the effect of BMI on mental well-being [10]. Self-esteem is another plausible mechanism in the psychology literature, and targeted mental health interventions are another potential policy tool to address these issues.

## Supporting information

S1 Fig Reduced form estimates. (DOCX)

S1 Table Beck’s Depression Inventory. (DOCX)

S2 Table Correlation between BMI and depression, 2017 and 2005. (DOCX)
