# Supplementary material for: Effect of weight on depression using multiple genetic instruments
Source: PLoS One. 2024 Feb 23;19(2):e0297594. doi: 10.1371/journal.pone.0297594 (PMC10889664; doi:10.1371/journal.pone.0297594)
Supplement: S1 Fig — (DOCX) [file pone.0297594.s001.docx]

S1 Fig. Reduced form estimates.


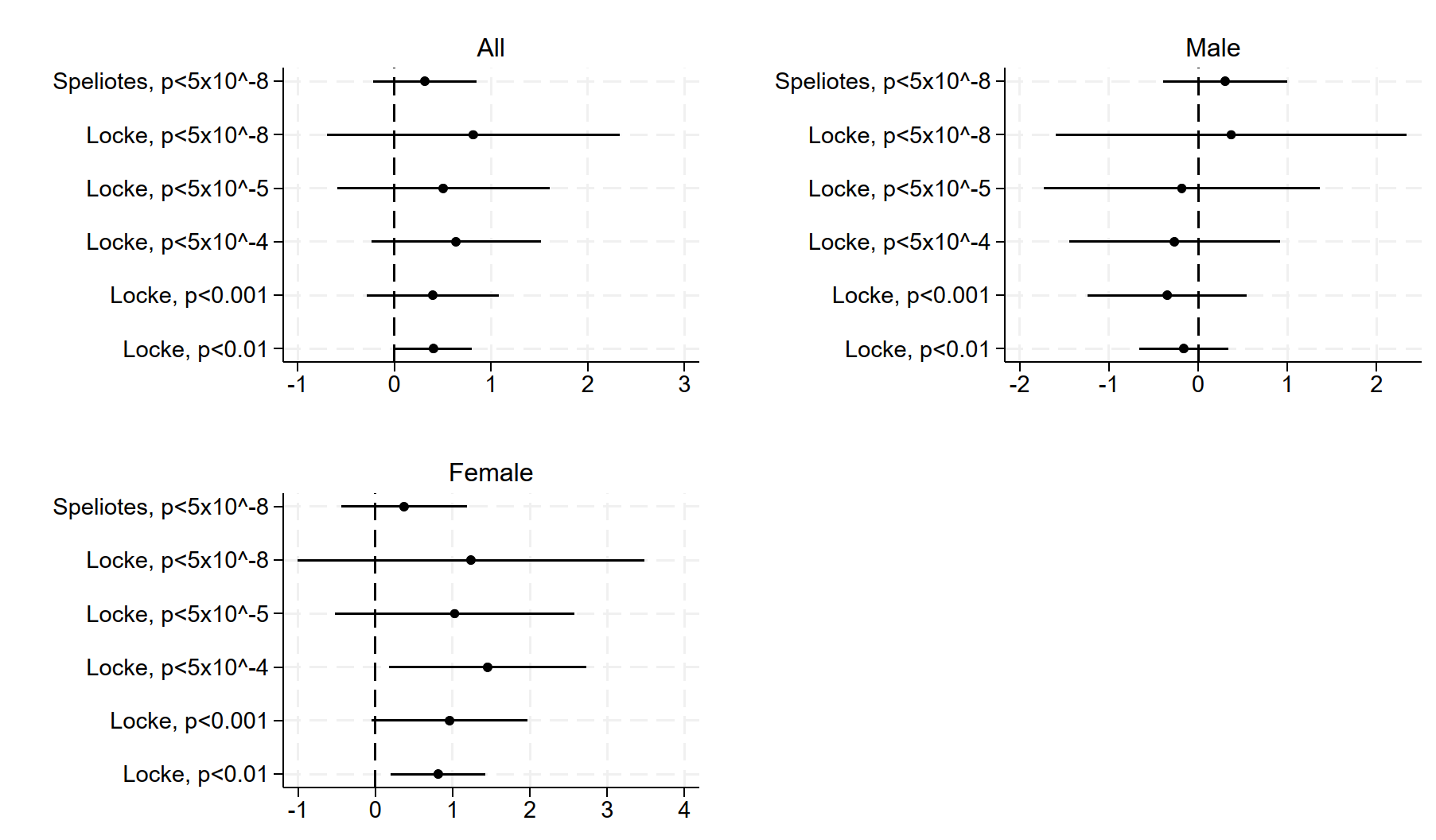


Note: The figure shows OLS point estimates for PGSs and the corresponding 95% confidence intervals based on heteroscedasticity-robust standard errors. The dependent variable is Beck’s Depression Inventory score measured in 2011. The models include controls for (sex), age, the first ten principal components, region of residence in 1980, and parental education. The PGSs are based on studies by Speliotes et al. (2010) and Locke et al. (2015). N = 1,523.

References

Locke AE, Kahali B, Berndt SI, Justice AE, Pers TH, Day FR, et al. Genetic studies of body mass index yield new insights for obesity biology. Nature. 2015;518:197–206. https://doi.org/10.1038/nature14177

Speliotes EK, Willer CJ, Berndt SI, Monda KL, Thorleifsson G, Jackson AU, et al. Association analyses of 249,796 individuals reveal 18 new loci associated with body mass index. Nat Genet. 2010;42(11):937–48. https://doi.org/10.1038/ng.686
