# Supplementary material for: Effect of weight on depression using multiple genetic instruments
Source: PLoS One. 2024 Feb 23;19(2):e0297594. doi: 10.1371/journal.pone.0297594 (PMC10889664; doi:10.1371/journal.pone.0297594)
Supplement: S1 Table — (DOCX) [file pone.0297594.s002.docx]

S1 Table. Beck’s Depression Inventory.

Q1.

0 = I do not feel sad.

1 = I feel sad.

2 = I am sad all the time and I can't snap out of it.

3 = I am so sad and unhappy that I can't stand it.

Q2.

0 = I am not particularly discouraged about the future.

1 = I feel discouraged about the future.

2 = I feel I have nothing to look forward to.

3 = I feel the future is hopeless and that things cannot improve.

Q3.

0 = I do not feel like a failure.

1 = I feel I have failed more than the average person.

2 = As I look back on my life, all I can see is a lot of failures.

3 = I feel I am a complete failure as a person.

Q4.

0 = I get as much satisfaction out of things as I used to.

1 = I don't enjoy things the way I used to.

2 = I don't get real satisfaction out of anything anymore.

3 = I am dissatisfied or bored with everything.

Q5.

0 = I don't feel particularly guilty.

1 = I often feel guilty about something I have done or failed to do.

2 = I feel quite guilty most of the time.

3 = I feel guilty all of the time.

Q6.

0 = I don't feel I am being punished.

1 = I feel I may be punished.

2 = I expect to be punished.

3 = I feel I am being punished.

Q7.

0 = I don't feel disappointed in myself.

1 = I am disappointed in myself.

2 = I am disgusted with myself.

3 = I hate myself.

Q8.

0 = I don't judge or blame myself any more than usual.

1 = I judge myself more than before.

2 = I blame myself for all my mistakes.

3 = I blame myself for everything that happens.

Q9.

0 = I don't have any thoughts of killing myself.

1 = I have thoughts of killing myself, but I would not carry them out.

2 = I would like to kill myself.

3 = I would kill myself if I had the chance.

Q10.

0 = I don't cry any more than usual.

1 = I cry more now than I used to.

2 = I cry for the smallest reasons.

3 = I would like to cry, but I can't do it.

Q11.

0 = I am not more restless or tense than usual.

1 = I am more restless or tense than usual.

2 = I am so agitated that it's hard for me to stay still.

3 = I am so agitated that I have to keep doing something all the time.

Q12.

0 = I am interested in other people and things as before.

1 = I am less interested in other people and things than before.

2 = I have lost almost all interest in people and things.

3 = Nothing interests me anymore.

Q13.

0 = I can make decisions as before.

1 = It is more difficult for me to make decisions than before.

2 = It is very difficult for me to make decisions.

3 = I can no longer make any decisions at all.

Q14.

0 = I don't consider myself worthless.

1 = I feel less needed than before.

2 = I am worth less than other people.

3 = I feel completely worthless.

Q15.

0 = I am as energetic as before.

1 = I have less energy than before.

2 = I don't have the energy to do much.

3 = I don't have the energy to do anything.

Q16.

0 = I sleep as well as before.

1 = I sleep a little more than before.

2 = I sleep a little less than before.

3 = I sleep a lot more than before.

4 = I sleep a lot less than before.

5 = I sleep most of the day.

6 = I wake up a couple of hours too early and can't get back to sleep.

Q17.

0 = I am not more irritable than before.

1 = I get irritated more easily than before.

2 = I get much more easily irritated than before.

3 = I am irritated all the time.

Q18.

0 = My appetite has not changed.

1 = I eat slightly less than before.

2 = I eat slightly more than before.

3 = I eat much less than before.

4 = I eat much more than before.

5 = I have no appetite at all anymore.

6 = I constantly crave food.

Q 19.

0 = I can concentrate as well as before.

1 = I can't concentrate as well as before.

2 = It is difficult for me to concentrate on one thing for long.

3 = I can't concentrate on anything.

Q20.

0 = I am not more tired than before.

1 = I get tired much more easily than before.

2 = I am too tired to do the things that should be done.

3 = I am so exhausted that I can't get anything done.

Q21.

0 = My interest in sex has not decreased recently.

1 = My interest in sex has decreased.

2 = My interest in sex is significantly less than before.

3 = I have completely lost interest in sex.

Q16 and Q18 were recoded as follows: 0 = 1, 1 = 1, 2 = 1, 3 = 2, 4 = 2, 5 = 3, and 6 = 3.
