# Supplementary material for: Effect of weight on depression using multiple genetic instruments
Source: PLoS One. 2024 Feb 23;19(2):e0297594. doi: 10.1371/journal.pone.0297594 (PMC10889664; doi:10.1371/journal.pone.0297594)
Supplement: S2 Table — (DOCX) [file pone.0297594.s003.docx]

S2 Table: Correlation between BMI and Depression, 2017 and 2005.

|  | Overall Depression | Overall Depression 2005 | Overall Depression 2017 | Average Depression | Average Depression 2005 | Average Depression 2017 |
| --- | --- | --- | --- | --- | --- | --- |
| BMI | 0.0022** | 0.0025* | 0.0020* | 0.0061*** | 0.0049*** | 0.0066*** |
|  | (0.0007) | (0.0011) | (0.0010) | (0.0006) | (0.0009) | (0.0009) |
|  |  |  |  |  |  |  |
| N | 6459 | 3141 | 3318 | 9820 | 4786 | 5034 |

Note: Standard errors in parentheses. *p < 0.05, ** p < 0.01, *** p < 0.001.

Results are from a bivariate regression on a measure of depression on BMI using data from the National Health and Nutrition Examination Survey. Overall Depression is based on the question “How difficult have these problems made it for you to do your work, take care of things at home, or get along with people?” Average Depression is based on the questions: “Over the last 2 weeks, how often have you been bothered by the following problems: little interest or pleasure in doing things? Would you say: *Have little interest in doing things?*”, “*Feeling down, depressed, or hopeless?*”, “*Trouble falling or staying asleep, or sleeping too much?”,* “*Feeling tired or having little energy?*”, “*Poor appetite or overeating?*”, “*Feeling bad about yourself - or that you are a failure or have let yourself or your family down?”,* “*Trouble concentrating on things, such as reading the newspaper or watching TV?”,* “M*oving or speaking so slowly that other people could have noticed? Or the opposite - being so fidgety or restless that you have been moving around a lot more than usual?”,* and “*Thoughts that you would be better off dead or of hurting yourself in some way?”*
